# Multifunctional Cu_2−x_Te Nanocubes Mediated Combination Therapy for Multi-Drug Resistant MDA MB 453

**DOI:** 10.1038/srep35961

**Published:** 2016-10-24

**Authors:** Aby Cheruvathoor Poulose, Srivani Veeranarayanan, M. Sheikh Mohamed, Rebeca Romero Aburto, Trevor Mitcham, Richard R. Bouchard, Pulickel M. Ajayan, Yasushi Sakamoto, Toru Maekawa, D. Sakthi Kumar

**Affiliations:** 1 Bio Nano Electronics Research Centre, Graduate School of Interdisciplinary New Science, Toyo University, Kawagoe, 350-8585, Japan; 2 Department of Imaging Physics, University of Texas MD Anderson Cancer Center, Houston, TX, 77054, USA; 3 Department of Material Science and Nano Engineering, Rice University, 6100 Main Street, Houston, TX, 77005, USA; 4 Biomedical Research Centre, Division of Analytical Science, Saitama Medical University, Saitama, 350-0495, Japan

## Abstract

Hypermethylated cancer populations are hard to treat due to their enhanced chemo-resistance, characterized by aberrant methylated DNA subunits. Herein, we report on invoking response from such a cancer lineage to chemotherapy utilizing multifunctional copper telluride (Cu_2−X_Te) nanocubes (NCs) as photothermal and photodynamic agents, leading to significant anticancer activity. The NCs additionally possessed photoacoustic and X-ray contrast imaging abilities that could serve in image-guided therapeutic studies.

Globally, basal-like breast cancers are documented to constitute 15–20% of all breast malignancies. They are characterized by aggressive cellular proliferation and short relapse times, culminating in poor patient prognosis. Breast cancers in general display a diverse range of biological, morphological and molecular subtypes defined by signature clinical characteristics. Basal-like breast cancers exhibit hyperactivity of DNA methylation as a result of DNA methyltransferase 3b (DNMT3b) overexpression, and simultaneous silencing of multiple methylation-sensitive genes. This typical feature resonates in the aggressive proliferation of basal-like breast cancer cells and translates in therapeutic resistance associated with them^1^,^2^,^3^,^4^,^5^,^6^,^7^. Therefore, it is crucial that strategies addressing and overcoming these challenges be developed at the earliest. It is imperative that the conventional therapeutic strategies be augmented significantly and accordingly, to deal with such complexities. With remarkably diverse physicochemical properties, nanomaterials have tremendous prospects in medical treatment options. The integration of multiple functionalities such as specific cell targeting, fluorescent monitoring, drug/biomolecule accommodation, signature signal based sensing etc., in a single nano-entity offers a plethora of opportunities in strengthening the current anti-cancer therapeutic regimes^8^,^9^,^10^,^11^,^12^,^13^,^14^,^15^,^16^,^17^,^18^,^19^. Inorganic nanoparticles as gold, metal sulfides etc., hold high-extinction coefficients in converting near-infrared (NIR) light to heat, and have been examined for photothermal therapy. Due to their tunable surface plasmon and NIR-thermal responsiveness, copper based chalcogenides seem promising in a wide range of biomedical applications and are ideally suited for theranostics^20^,^21^,^22^. Being p-type semiconductors, copper based chalcogenides determine charge transport and provide a composition-dependent localized surface plasmon resonance (LSPR) in the near-infrared (NIR) region^23^,^24^,^25^,^26^. In a recent work, Li *et al.* for the first time demonstrated the possibility of using as-made CuTe nanocrystals as a new NIR absorbing agent for *in vitro*-photothermal-killing of cancer cells^27^. However, only a limited number of reports exist on Cu_2_Te, demonstrating its multifunctional theranostic capabilities. Herein, we developed size and shape tunable multifunctional Cu_2−X_Te nanocubes that presents a strong absorbance in the NIR region, ideal for photoacoustic (PAI) and X-ray contrast imaging. The nanocubes serve as highly effective chemo-photothermal-photodynamic cancer therapeutic combinatorial treatment agents that can overcome hypermethylated cancer cell resistance to chemotherapeutic drugs.

## Results and Discussion

### Nanocubes synthesis and characterization

Copper telluride NCs were prepared by reacting Cu (I) Cl with tri-n-butylphosphine telluride in the presence of diphenylphosphine (DPP), and octadecene. In a typical synthesis, 0.6 mmol of Cu (I) Cl was mixed with 15 mmol of DPP and 10 mL of octadecene in a 100 mL three-neck flask. The mixture was heated under vacuum to 100 °C to obtain a clear solution and kept at this temperature for 60 min to remove low-boiling impurities. The temperature was then increased to 160 °C. Meanwhile, a tellurium precursor solution was prepared by mixing 0.3 mmol Te powder with 0.5 mL of tri-n-butylphosphine solution, heating the mixture to 200 °C. Post cooling the tellurium precursor solution to room temperature, the solution was swiftly infused into copper solution maintained at 160 °C. The solution color immediately changed to dark brown or green. The reaction was stopped after 45 min and the NCs were precipitated and washed using excess ethanol after repeated centrifugation at 8000 rpm. The obtained precipitate was dispersed in chloroform until further processing. Besides assisting Cu salt dissolution, we believe that DPP aids in anisotropic growth of the NCs due to their bulky phenyl groups, which prevent the growth of NCs in certain phases favoring cubic nanostructure formation. It is also speculated that these phenyl groups facilitate the stabilization of surface of the NCs during the crystal growth, thereby producing cubic shaped, size tuned NCs. The reaction time, temperature and concentration of DPP were key parameters to control the size and shape of copper telluride NPs obtained.

To assess the effects of reaction times on the synthesis of NCs, a 2–60 min variation in reaction time was exercised. At shorter reaction times, 2 and 10 min, few NCs were formed, however not uniform in size or shape. At reaction time ranging from 30–60 min, the formation of NCs was observed, which was confirmed by their NIR absorption (Figure S1). In the 30–60 minute experimental window (30, 45 and 60 min), the reaction at 45 min yielded better cubic nanostructures (Figure S2) with good NIR absorption. Investigating the role of temperature on NCs synthesis, it was found that lower temperature synthesis yielded poorly formed NCs whereas high temperature resulted in aggregation. It was inferred from the poor formation of NCs below 150 °C that higher temperature is crucial for nucleation process. Synthesis at 160 °C yielded size and shape tunable cubic nanostructures with better NIR absorption. The DPP concentration was optimized based on the solubility it rendered to 0.6 mmol Cu salts, a concentration of 15 mmol was found to be optimum. Therefore the reaction was optimized at 160 °C for 45 min with 15 mmol of DPP and the resultant NCs were employed in further characterization and application.

**Figure 1:**
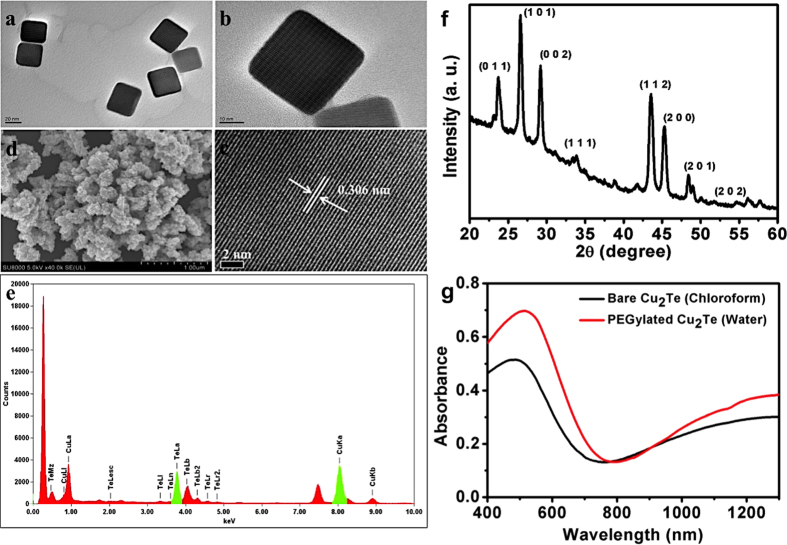
Structural, elemental, crystalline and optical characterization of the as synthesized copper telluride NCs. (**a**,**b**) Representative TEM images depicting the cuboid morphology of the NCs (**c**). HR-TEM image exhibiting the crystal lattice distance, (**d**). SEM image of the NCs, (**e**). EDS analysis of the NCs confirming the presence of Cu and Te, without any other elemental impurities. (**f**) XRD analysis of the NCs confirming that the crystal phases of the synthesized NCs depicts the rickardite crystalline pattern (JCPDS 01-089-2127) and (**g**). UV-Vis-NIR absorption spectra of bare and PEGylated NCs displaying a clear NIR absorption onset.

Figure 1a–c shows representative transmission electron microscopy (TEM) and high-resolution TEM (HRTEM) micrographs of the copper telluride NCs. A cubic geometry with narrow size distribution of 35–40 nm was observed (Fig. 1a), which facilitated the self-assembly into cubic superlattices. HR-TEM confirms the high crystallinity of NCs with crystal lattice fringes spaced at 0.306 nm, matching the 002 plane of rickardite (Fig. 1c), JCPDS 01-089-2127. The monodispersity of NCs and their smooth surface morphology was observed under SEM (Fig. 1d). Energy Dispersive X-Ray Analysis (EDS) analysis confirmed the purity of sample with Cu and Te in an atomic ratio of 1.6:1 (Fig. 1e). X-Ray diffraction (XRD) analysis of the NCs exhibited sharp peaks indexed to be tetragonal rickardite crystal structure (JCPDS 01-089-2127) (Fig. 1f). Study of optical property of the NCs, using UV-Vis-NIR absorption spectroscopy, exhibited an absorption onset at 600 nm and a broad, strong absorption centered at 1250 nm associated with LSPR in the NIR region (Fig. 1g) due to the presence of minority carriers that arise from Cu deficiencies in the NCs.

**Figure 2:**
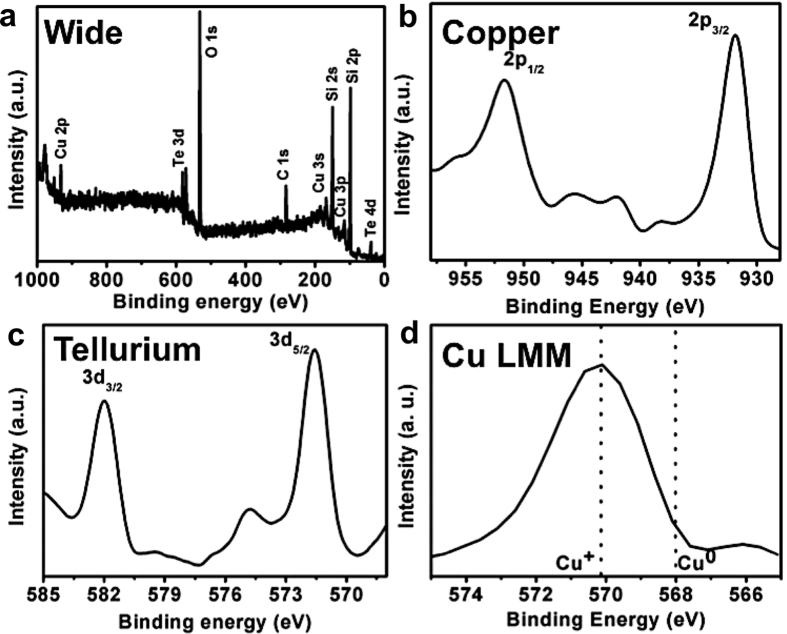
XPS analysis of the NCs showing spectrum of wide, Cu 2p, Te 3d and Cu LMM peak. Two distinct peaks at 931.83 eV and 951.65 eV were assigned to Cu 2p_3/2_ and Cu 2p_1/2_ confirming Cu^+^ oxidation state of Cu. The peaks at binding energies of 571.56 and 581.86 eV were consistent with Te 3d_5/2_ and 3d_3/2_ arising from Te^2−^ oxidation state. The actual oxidation state of Cu was found to be Cu^+^1 by analyzing Cu LMM peak.

X-Ray photoelectron spectroscopy (XPS) was carried out to analyze the oxidation states of Cu and Te (Fig. 2). Two distinct peaks at 931.83 eV and 951.65 eV were assigned to Cu 2p_3/2_ and Cu 2p_1/2_ with a spin-orbit separation of 19.82 eV, respectively. The peaks of Cu 2p confirm Cu^+^ oxidation state of Cu, with full width half maxima of Cu 2p_3/2_ and Cu 2p_1/2_ to be 2.91 and 3.99, respectively. In the case of Te, peaks at binding energies of 571.56 and 581.86 eV were consistent with Te 3d_5/2_ and 3d_3/2_ arising from Te^2−^ oxidation state. The Te 3d_5/2_ and 3d_3/2_ exhibit a spin-orbit separation of 10.3 eV with FWHM of 1.72 and 1.66, respectively. However, slight shoulder satellite peaks corresponding to other oxidation states of Cu and Te were also noticed, though these were comparatively lower in intensity to Cu^+^ and Te^2−^. The intensity of satellite peak correlating to Cu^2+^ oxidation state was found to be 12%, whereas the predominant Cu^+^ peak exhibited an intensity of 87.2%. The presence of other oxidation states could be attributed either to the partially filled d-shell in transition metals that allows for the adoption of multiple oxidation states or to the slight oxidation due to atmospheric exposure. Subsequent investigation of the actual oxidation state of Cu by analyzing Cu LMM (Fig. 2d) peak revealed the oxidation peak of Cu to be +1.

Hydrophilicity of the NCs is a principally crucial aspect when considering a nanomaterial for biological applications. To address this issue, a biofunctionalization, utilizing lipid-PEG hybrids to cap the NCs via a simple thin film hydration technique was employed^21^. The NCs were desiccated completely with lipid-PEG resulting in a thin film, which was further hydrated using PBS, yielding lipid-PEG coated NCs (PEG-NCs). The hydrodynamic diameter of the PEG-NCs was found to be 199.6 nm (Figure S3) whereas the zeta potential was −41.4 mV (Figure S4). The Fourier transform infrared spectroscopy (FT-IR) analysis of the PEG-NCs displayed signature peaks of PEG-lipid grafts at 1058 (C–O–C ether stretch band), 2918 (–CH_2_ stretching vibrations) and 942 cm^−1^ (–CH out-of-plane bending vibrations), confirming successful PEGylation (Figure S5). Post PEGylation, the NIR absorption of PEG-NCs were analyzed and were found to be unaffected by the biofunctionalization step. The overall LSPR absorption property of the NCs in the NIR region was completely retained post-PEGylation depicting the successful biofunctionalization without altering the intrinsic optical nature of NCs (Fig. 1g).

### PAI and X-Ray contrast evaluation

**Figure 3:**
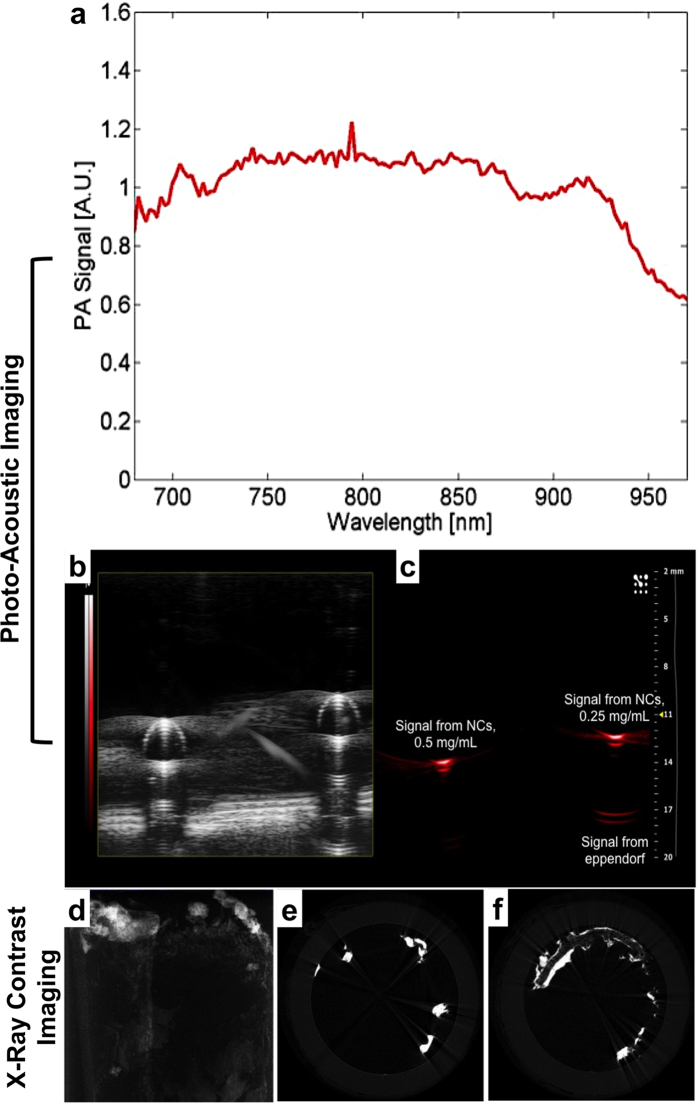
Multimodal imaging abilities of the NCs. (**a**–**c**) Photoacoustic imaging. Normalized photoacoustic signal spectrum of the NCs (left) and photoacoustic image (right) of PEGylated NCs filled eppendorf obtained with the VisualSonics Vevo 2100-LAZR system. The gray scale image in the right panel displays that the NCs possess no contrast under ultrasound imaging module. (**d**–**f**) X-ray microCT imaging of the NCs at tube voltage of 45 KeV. Cross sectional X-ray contrast imaging of paper phantom with NCs (left). The Z-stacked contrast image of the NCs is shown in (**e**). First image of z-stacking and (**f**). Corresponds to the 100^th^ image of z-stacking.

Next, we investigated the PAI ability of PEG-NCs in the NIR region (Fig. 3a–c). The spectroscopic imaging of NCs generated a broad photoacoustic signal denoted by the absorption around 900 nm under preclinical frequency (21 MHz); however the NCs failed to produce ultrasound contrast. Subsequently, we examined the ability of PEG-NCs to perform as X-ray attenuation based contrast agents. Elements with atomic numbers higher than of body tissue elements (C, O, N, P etc.) produce contrast when exposed to X-rays. Figure 3d–f shows the X-ray computed tomography (CT) image of PEG-NCs and their cross-sectional Z-stacks exhibiting evident signal enhancement, demonstrating the potential application of the NCs as CT contrast agent. Though the CT contrast exhibited by metal nanostructures are not on-par with the commercial ones and are preliminary, we believe the ability can be improvised with doping the nanostructures with other elements such as Au, Bi etc.

### NIR induced photothermal conversion

**Figure 4:**
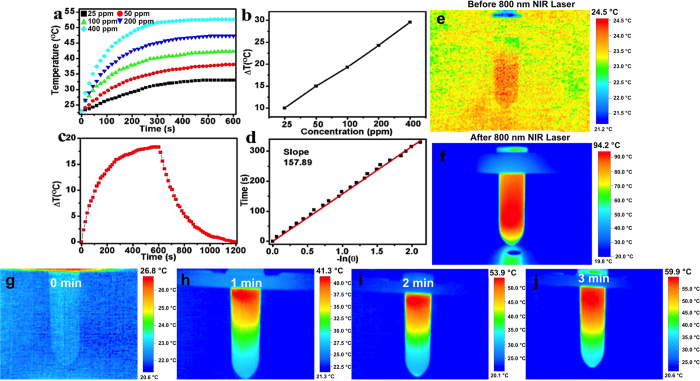
Photothermal efficacy analysis of the NCs. (**a**) Heating profile of different concentrations (25, 50, 100, 200 and 400 ppm) of PEGylated NCs (suspended in water) upon NIR irradiation. (**b**) Plot of difference in temperature attained upon 600 sec (10 min) NIR irradiation of different concentrations of NCs. (**c**) The heating and cooling profile of 100 ppm NCs in terms of difference in temperature. (**d**) Plot of cooling period (after 600 s) versus negative natural logarithm of driving force temperature. Time constant (τs) for heat transfer is determined to be 157.89 s. (**e**,**f**) Temperature rise from 24.5 °C to 94 °C within 10 sec of NIR irradiation observed with non-PEGylated bare NCs solution. (**g**–**j**) Temperature rise from 26.8 °C to 59 °C was recorded post 3 min of NIR irradiation with PEGylated NCs solution.

The human tissue is transparent to NIR laser and thus NIR laser reliant photothermal therapy holds selective efficacy over conventional treatment options. A number of nanomaterials are known to serve as efficient NIR based photothermal conversion agents^28^,^29^,^30^,^31^, especially carbon and metal based nanostructures that have found extensive interest in PTT appications^32^,^33^,^34^,^35^. To verify the potential of NC’s as PTT agents, different concentrations of PEG-NCs solutions were irradiated with 808 nm laser at a constant laser power, 2027 mW/cm^2^. The heating of PEG-NCs solution increased with concentration, indicating the linear relationship between temperature elevation and NCs concentration. During the irradiation of PEG-NCs at concentrations of 25, 50, 100, 200 and 400 ppm for 10 min (Fig. 4a), the 25 ppm solution caused a temperature rise of ~10 °C from the initial temperature, which is much higher than that of pure water. The change in temperature of 400 ppm PEG-NC solution was found to rise 28 °C higher than that of initial temperature on laser irradiation for 10 min (Fig. 4b). These observations positively affirmed the conversion of NIR laser energy into heat by PEG-NCs. It is established that the temperature maintained at 42~45 °C for about half an hour cause irreversible cellular damage and cell death. Therefore, the PEG-NCs-NIR laser induced temperature increase could potentially be used to initiate irreversible damage to cancer cells and/or tissues. Subsequently, we measured the photothermal conversion efficiency (PCE) of PEG-NCs by studying the heating and cooling profile of the NCs (100 ppm) (Fig. 4c). The plot of cooling period versus negative log of temperature and time constant for heat transfer was determined (Fig. 4d). Based on these data, the photothermal conversion efficiency of PEG-NCs was calculated and found to be 25.68%, a commendable and comparable efficiency value in relation to Au NPs. A thermograph observation revealed that in the case of bare (non-PEGylated NCs), within 10 sec of NIR irradiation the temperature rose to >90 °C (Fig. 4f) whereas the PEG-NCs reached a maximum temperature of >55 °C after 3 min of NIR laser irradiation (Fig. 4j). This decrease in the temperature rise could be attributed to the PEG-lipid coating over the NCs. Though there was a marked decrease observed in the heat generation on NIR laser exposure, as mentioned earlier, constant temperature maintenance in excess of 42 °C is enough to cause severe lethality to the cells under exposure.

### NIR induced photodynamic property

Nanoparticle-based photodynamic therapy (PDT) is potentially useful to significantly improve the performance of therapy by overcoming limitations of organic dyes that are in use currently. Nanomaterials able to induce formation of singlet oxygen, and exert PDT effects upon light exposure are very rare^36^,^37^,^38^. Recently, the intrinsic ability of gold nanorods (Au NRs)^38^, Cu_2−x_S^23^, C_60_^39^ and W_18_O_49_ nanowires^40^ to serve as NIR light responsive PDT agents were reported; however, not much literature is available about the intrinsic PDT abilities of other nanostructures. Cu_2−X_Te, has good absorption in NIR region; and an attempt at investigating their PDT performance was initiated based on DCFH-DA fluorescence assay^23^. The NIR irradiation of aqueous solution of copper telluride leads to leaching of Cu ions from the NCs, possibly enhancing the generation of reactive oxygen species (ROS). This effect is highly specific as it occurs only upon irradiation and scales with temperature. The generation of ROS was tracked with the DCFH-DA, a well-known oxidation-sensitive fluorescent dye. When the NCs were irradiated, ROS in the aqueous solution converts the DCFH-DA into a fluorescent form that is measured spectrophotometrically. Water (non-NIR irradiated & irradiated) and NC suspension without NIR irradiation failed to generate ROS, and thus no fluorescence was quantified. However, the NCs when exposed to NIR generated excess ROS and thus the fluorescent emission of DCFH-DA (Figure S6). This experiment confirmed the ability of PEG-NCs to produce ROS on NIR laser irradiation. The ample ROS that is produced upon irradiating NCs, can oxidize bio-macromolecules, cause cell damage and apoptosis.

### Cytocompatibility of Cu_2_Te nanocubes

**Figure 5:**
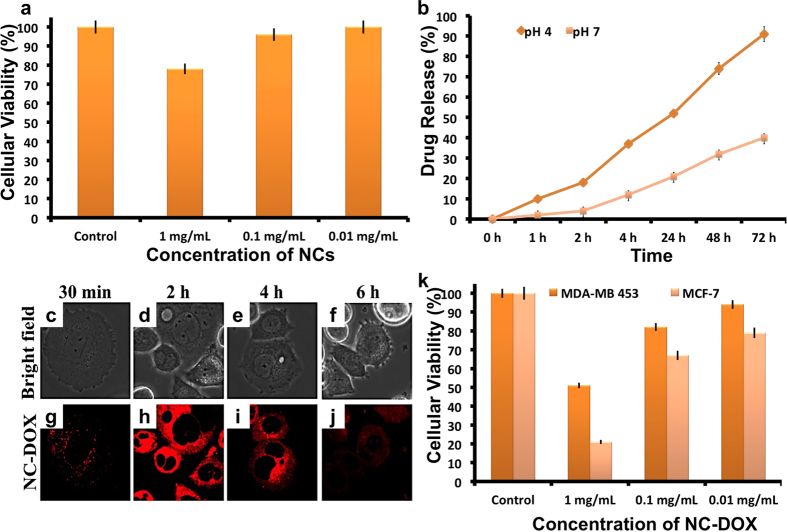
Biocompatibility analysis of the NCs and their drug release pattern intracellularly. (**a**) *In vitro* viability analysis of cells exposed to PEGylated NCs at different concentrations (0.01–1 mg/mL) post 72 h. (**b**) Acidic pH dependent DOX release from PEG-NC-DOX conjugate. (**c**) Cellular entry analysis of PEG-NC-DOX conjugate confirmed the entry of NCs into the cell’s (MDA-MB 453) cytoplasm (DOX’s fluorescence). The images are acquired at different time points (0.5–6 h) post NC exposure. (**d**) *In vitro* cancer killing ability of PEG-NCs-DOX at different concentrations (0.01–1 mg/mL) measured with the help of alamar blue assay. MCF-7 is found to be susceptible to nano-chemotherapeutic whereas MDA MB 453 is not. Error bars indicates SE.

Next, we analyzed the intrinsic toxicity of the NCs, as toxicity studies are of prime importance before employing any material, nano or otherwise for bio-applications. In this context, the viability of PEG-Cu_2−X_Te treated hypermethylated MDA MB 453 breast cancer cells was assayed. PEG-NCs at concentrations of 1, 0.1, 0.01 mg/mL were exposed to cells for 72 h and their metabolic activity analyzed (Fig. 5a). A slight dose dependent reduction in cellular viability was observed with the highest concentration, 1 mg/mL registering a viability of approximately 78%, which is not a drastic deviation from control values. The commendable cellular viability attests the safety of these NCs for further biological processing.

### DOX loading and release

Apart from their signature properties, nanomaterials play an important role as cargo carriers^41^,^42^,^43^,^44^. Due to their high surface area they can transport relatively higher concentration of drugs/peptides/nucleic acid etc. to the target site more effectively^45^,^46^,^47^. In addition, due to their nanoscale size, they can trespass biological barriers with high efficiency resulting in increased beneficial therapeutic outcome^48^. Herein, to validate the effect of combined chemo-photothermal therapy, doxorubicin (DOX) was adsorbed onto the PEG-NCs. DOX, being a hydrophobic drug, specifically interacts with hydrophobic lipid chains of lipid-PEG hybrid NCs by simple hydrophobic-hydrophobic interaction. Drug attachment was confirmed by photoluminescence (PL) emission of DOX from the DOX-NCs conjugate (Figure S7) and the loading efficiency was found to be approximately 79%. DOX release from the NCs was observed to be highly pH dependent, as nearly 80% got released by 48 h at pH 4, whereas the release at pH 7 was about 30% (Fig. 5b). This observation can be ascribed to the amine groups present in DOX that protonate at low pH favoring a faster release. This acidic pH dependent drug release prevents premature release of the drugs at physiological pH, facilitating safe delivery of maximum therapeutic cargo specifically to the targeted cancer cells (internal pH of cancer cells is known to be acidic compared to normal cells).

### *In vitro* cellular imaging and chemo-toxicity of NCs-DOX conjugate

The NCs mediated drug delivery to cancer cells was further recorded with the cells being exposed to DOX-PEG-NCs for 2 h, followed by imaging of cells using the intrinsic fluorescence of DOX when excited at 561 nm. Though the NCs themselves lacked the optical imaging characteristic, the presence of DOX assisted to monitor the localization of DOX-PEG-NCs (Fig. 5c–j). DOX-PEG-NCs principally amassed in the endosomal vesicles within 30 min of exposure and the fluorescence was localized in the cytosol even after 4 h, inferring the stable attachment of the drug to NCs. The usual translocation of DOX into nuclear spaces that occurs in most cancer cells by 6 h after internalization was not observed here. By 6 h, we found a gradual decrease in fluorescence intensity of DOX-PEG-NCs in endo/lysosomal vesicles. We believe that the loss of DOX is due to the presence of multi-drug resistance (MDR) channels in hypermethylated MDA MB 453 cells that effectively pumps out the drug. When a comparative study on another breast cancer cell line, MCF-7 (a non-hypermethylated) was done with DOX-PEG-NCs, DOX was found to be accumulated in endosomes by 30 mins and later accumulated in nucleus for an extended period (Figure S8). This comparative analysis affirmed the inability of the present nano-chemotherapeutics to reach their site of action, the nucleus, in hypermethylated cells due to the drug resistance and efflux protein machinery these cells possess. When the cytotoxicity rendered by DOX-PEG-NCs to MDA MB 453 cells was analyzed, considerable cell viability, approximately 51%, in 1 mg/mL DOX-PEG-NCs group (Fig. 5k) was noted. MCF-7 (luminal cancer) on the other hand, was found to be significantly sensitive to DOX-PEG-NCs at a concentration of 1 mg/mL registering only 21% cell viability (the IC_50_ of DOX to MDA MB 453 and MCF-7 cells was found to be 0.082 and 0.015 mg/mL respectively; the IC_50_ of MDA MB 453 cells was five times higher than that of MCF-7 cells which confirms the drug resistant/efflux nature of MDA MB 453). Phase contrast microscopic analysis provided a visual confirmation of the MCF-7’s mortality features, whereas the MDA MB 453 stayed viable and intact. Cancers, as resistant as MDA MB 453, demand high concentration of chemotherapeutics to kill them, which lead to undesired and non-specific toxicological side effects. Although the nano mediated DOX delivery (DOX-PEG-NCs), exhibited higher cytotoxic indices than that of DOX alone, it was obvious that they cannot exterminate cancer cells completely.

### NIR induced cellular photothermal ablation

**Figure 6:**
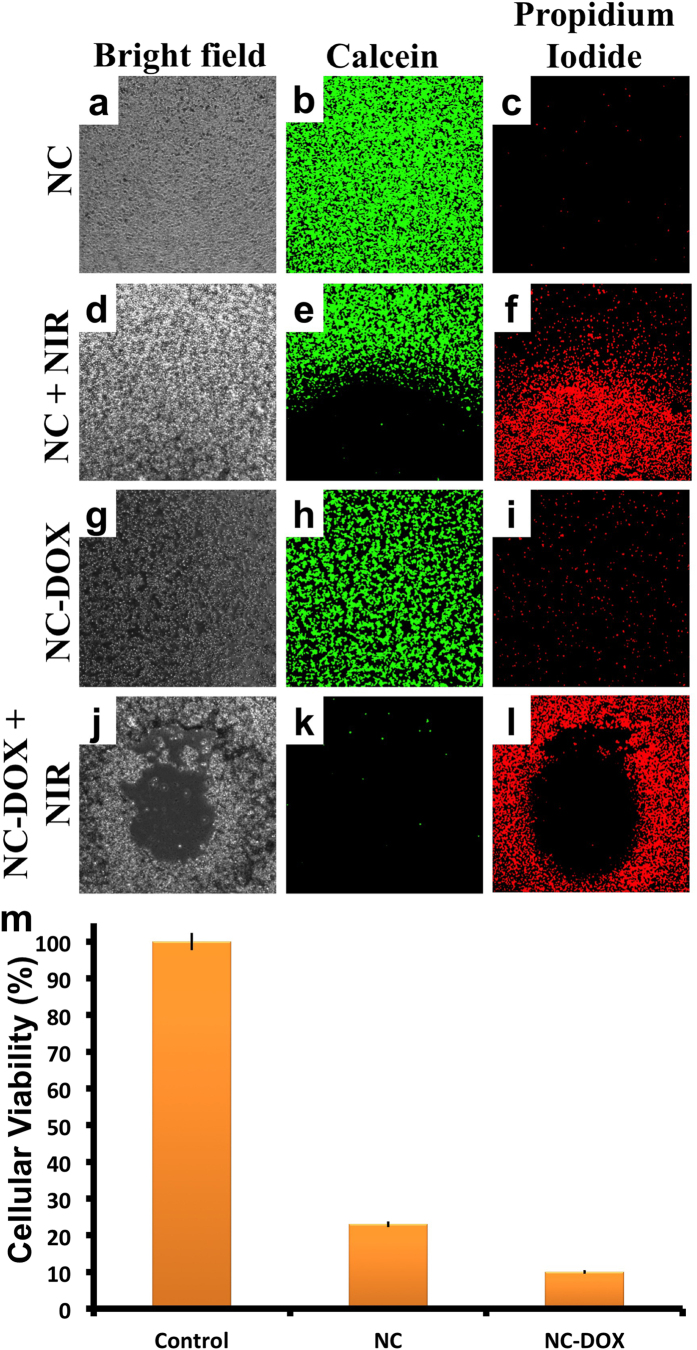
(**a**–**l**) Photothermal ablation of cancer cells (MDA-MB 453) by dual synergistic effect of drug and heat (induced by NIR irradiation), post calcein/PPi staining. Calcein maps live cells esterase with green fluorescence whereas the dead cells were stained red with propidium iodide’s dead cell specific membrane permeability. (**m**) *In vitro* anti-cancer activity of photothermal therapy (NC concentration: 0.2 mg/mL) to MDA-MB 453 cells measured with alamar blue assay. The error bar indicates SE.

Next, to test the hypothesis of sensitizing and killing such hypermethylated cancers by photothermal or synergistic photothermal-chemo therapy, NIR laser irradiation was employed, to which the NCs had shown excellent responsiveness. The results, post NIR irradiation were recorded qualitatively using live/dead cell tracker calcein/propidium iodide (Fig. 6a–l). Cells exposed to DOX-PEG-NCs displayed a distinct demarcation between the dead (red) and live (green) cells. Cells exposed to PEG-NCs (0.2 mg/mL) followed by 10 min NIR irradiation registered 23% viability when analyzed at 24 h post NIR exposure (Fig. 6m). However, when NIR irradiation of cells exposed to DOX-PEG-NCs (0.2 mg/mL) was analyzed, only 10% viability was recorded. These observations positively support the synergistic effect of photothermal therapy combined with chemotherapy to successfully eliminate multi-drug resistant, hypermethylated cancer cells. It is believed that increase in temperature sensitizes the cancer cells to chemotherapeutics, thereby killing them efficiently. Also, the high temperature attained during PTT induces the expression of heat shock proteins that negatively affects the plasma membrane integrity of the cancer cells. Due to loss of cell membrane permeability, cancer cells become more susceptible to DOX as their drug-efflux is affected, leading to effectual cell death. It is evident with the experimental observations that NC mediated PTT alone or in combination with chemodrugs can serve as better treatment strategy for hypermethylated chemo-resistant cancer cell types.

### NIR induced cellular photodynamic therapy

**Figure 7:**
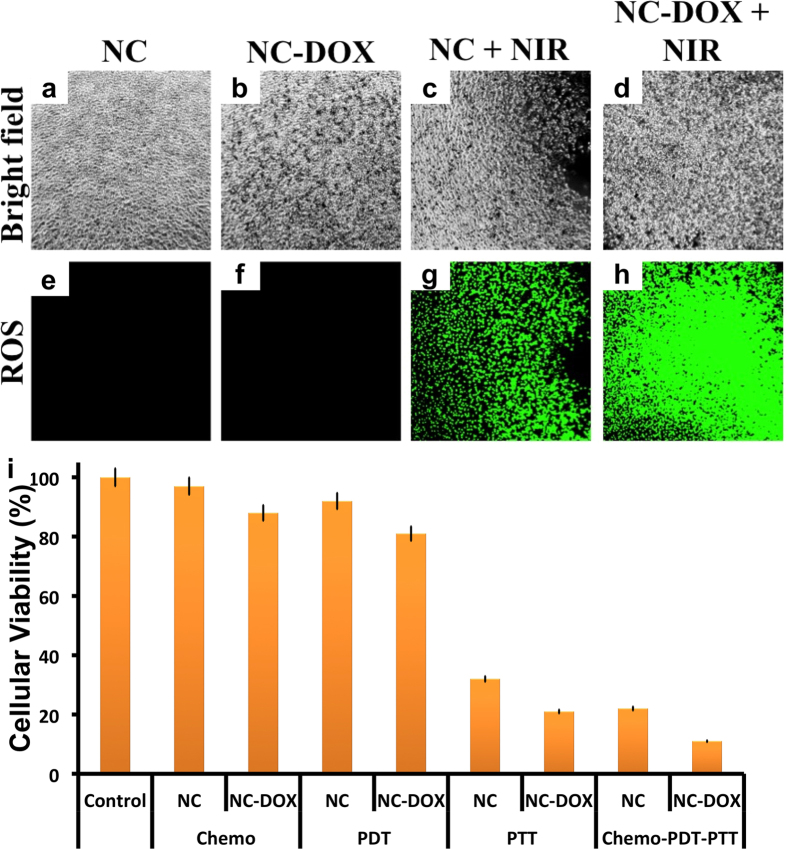
(**a**–**h**) Photodynamic therapy of cancer cells (MDA-MB 453) by dual synergistic effect of drug and ROS. The ROS produced post NIR irradiations were mapped with the help of Cell-ROX tracer, a non-fluorescent dye in the absence of ROS, that becomes fluorescent when it comes in contact with cellular ROS (**i**) Synergistic (MDA-MB 453) activity of PEG-NC/PEG-NCs-DOX (NC concentration: 0.05 mg/mL) by employing Chemo-Photothermal-Photodynamic therapy simultaneously measured with the help of alamar blue assay. Error bar indicates SE.

To further test the multimodal potential of the Cu_2−X_Te NCs, their role in photodynamic therapy was evaluated. MDA MB 453 cells were exposed to NCs at very low concentration (0.05 mg/mL), as this concentration does not increase the temperature rapidly upon NIR radiation, however can efficiently yield ROS. Cells exposed to NCs/NCs-DOX irradiated with NIR for 2 min, exhibited bright fluorescence in their cytosol when treated with intracellular ROS tracer (Fig. 7a–h). To track whether the produced ROS can induce cancer cell apoptosis, we analyzed the viability of cells post 24 h of NIR irradiation. The cell viability dropped to 92% (PEG-NC treated cells), whereas the cells treated with NC-drug conjugate, exhibited 81% viable cells post 24 h of NIR irradiation (Fig. 7i). The results depict a better cell death with NC-DOX conjugate with PDT when compared to chemo alone (88% cells viable when treated with 0.05 mg/mL of NC-DOX conjugate). However, when the NIR irradiation was prolonged (10 min, temperature ≤ 42 °C; PTT), nearly 79% of cells treated with NC-DOX (0.05 mg/mL) were dead.

### Synergistic combinatorial therapy

**Figure 8:**
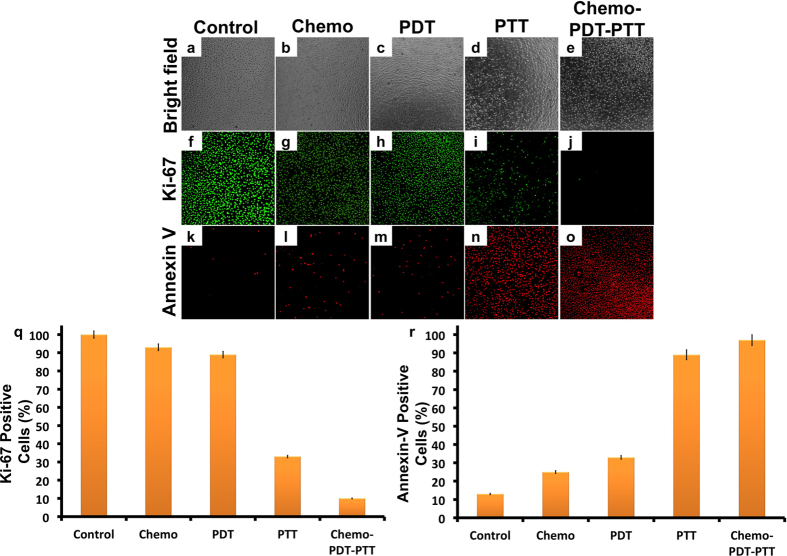
Combinatorial therapeutic approach (Chemo-PDT-PTT). The therapeutic nanoformulations at a concentration of 0.05 mg/mL for chemo (PEG-NCs-DOX), PDT (PEG-NCs-DOX + 2 min laser exposure) and PTT (PEG-NCs-DOX + 10 min laser exposure) exhibited reduced cell proliferation (Ki-67 panel in a; b) and increased apoptotic signals (Annexin-V panel in a; c) when compared to controls. Cell proliferation was evaluated after immunofluorescent staining with Ki-67 marker whereas the apoptosis was analyzed using Annexin-V. Error bars indicate SE.

Synergistic combinational therapy with modules like chemo, PTT and PDT is usually attained by hybrid/core-shell/manually assembled nanostructures that carry drug cargoes^49^,^50^. Not many reports are available when it comes to use of a single NC, with intrinsic abilities to serve imaging cum multiple therapeutic options such as PDT and PTT. Carbon nanohybrid structures that consist of graphene and C_60_ units were reported to serve as PDT and PTT agents^51^. Recently multi-branched gold nanostructures were used as NIR light activated PDT and PTT agent in second biological window^52^. Thus, to understand the role of synergistic therapy, we performed an experiment where cells were first exposed to NC-DOX (for 4 h) then subjected to PDT (irradiated for 2 min). Post irradiation, the cells were incubated for 4 h at ambient growth conditions after which, the cells were again irradiated for PTT (10 min) and then incubated for 16 h at ambient growth conditions. After the incubation period, the viability of cells were measured and found to be 11% (Fig. 7i). The viability with chemo-PDT-PTT in synergy was 11% whereas cells exposed to chemo-PTT registered 21% proving the efficacy of combination synergistic multimodal therapy. Next, we analyzed the proliferation ability of the cells post single/synergistic therapy. The proliferation ability of the cells were slightly compromised with chemo and PDT treatments, however with PTT and synergistic therapy the proliferative ability was completely diminished with very few cells exhibiting positive Ki-67 signals (Fig. 8f–j,q). A majority of cells that were exposed to PTT and synergistic therapy exhibited clear apoptotic signals indicative of actuated apoptotic cell death pathway (Fig. 8k–o,r).

Though PTT alone seemed to be an efficient strategy, the cells post PTT recover from the heat shock and their count increased after 72 h. However when a combinatorial therapy is administered, any such increase in cell count was observed. It is believed that the remnant cells due to the heat shock become susceptible to the synergistic effect of DOX and internally produced ROS, culminating in cell death. In addition, the combinatorial therapy brings down the amount of drug and NCs required to achieve the end results. When developing a therapeutic system against drug-resistant cancer cells, it has to be acknowledged that the efflux system of these cells can effectively pump out the drugs that can in turn act upon adjacent normal cells leading to significant side effects.

With PTT, the NC load, though can be decreased dramatically, still a few cells recovered from the heat shock and continued to propagate. It is also to be noted that 1 mg/mL of NC-DOX was required to kill the cancer cells (chemotherapy alone) whereas with combinatorial approach (chemo, PTT and PDT), the NC-DOX load was minimal (0.05 mg/mL) with improvised therapeutic outcome. We believe that the intrinsic multiple abilities of Cu_2−X_Te NCs to transport drug, produce ROS and heat upon NIR irradiation helps to overcome the highly resistant cancer type.

## Conclusion

Herein we propose an efficient module with which such cancer cells can be curbed even if they stay highly resistant to chemo/photothermal/radiation/hormonal therapy by combinatorial therapy using nanotheranostic Cu_2−X_Te NCs. These results demonstrate that combinatorial therapy can be an excellent technique for development of rational therapeutic approaches for hypermethylated breast cancers. We provide a proof-of-concept that multifunctional Cu_2−X_Te NCs can serve as sensitizing agents for treatment of hypermethylated breast cancer cells.

## Materials and Methods

### Materials

Cu (I) Cl, diphenylphosphine (DPP), chloroform, ethanol, dimethylsulfoxide were purchased from Kanto chemicals, Japan. Octadecene and tri-n-butyl phosphine (TBP) were purchased from Tokyo chemical industry, Japan. Te powder, doxorubicin hydrochloride, DCFH-DA and live/dead cell double staining kit were purchased from Aldrich. 1,2-distearoyl-sn-glycero-3-phosphoethanolamine-N-[amino(polyethylene glycol)-2000] (ammonium salt) (DSPE-PEG-Amine) was from Avanti Polar Lipids, Alabama. Alamar blue, Alexafluor Annexin-V 561 and ROS tracer were from Invitrogen. Anti Ki-67 antibody and its secondary antibody were purchased from abcam. All chemicals and reagents were of analytical grade.

### Synthesis of Cu_2−X_Te NCs

0.6 mmol of Cu (I) Cl dissolved in 10 mL octadecene (ODE) and 15 mmol DPP was first heated to 100 °C under vacuum for 30 min. Post 30 min, the temperature was raised to 160 °C and maintained under Ar atmosphere. 0.3 mmol of Te powder dissolved in 0.5 mL TBP was injected into the above-prepared solution. The reaction was maintained at 160 °C for 45 min. After 45 min, the reaction vessel was cooled to room temperature in an ice bath and the synthesized NCs were centrifuged and washed with ethanol several times. The final precipitate was re-dispersed in chloroform and stored at room temperature.

### Characterization and Instrumentation

The morphology of as prepared Cu_2−X_Te NCs was analyzed with field emission transmission electron microscope (TEM), (JEOL JEM-2100) and scanning electron microscope (Hitachi SU8020). The elemental composition of the NCs was analyzed using EDS (JEOL JED-2300T). Zeta potential and hydrodynamic size of the NCs was determined by Nano-ZS 168 Zetasizer (Malvern Instruments Ltd). Fourier transform infrared spectroscopy was performed with the help of Spectrum 100 FT-IR Spectrometer (Perkin Elmer). The optical property of the Cu_2−X_Te NCs was analyzed using a UV-Vis-NIR spectroscope (JASCO V-570 UV/Vis/NIR spectrophotometer). Photoluminescence spectra of Cu_2−X_Te-DOX NCs were recorded with excitation wavelength 365 nm using JASCO FP 6500 spectrofluorometer. X-ray photoelectron spectroscopy (XPS) measurement was carried out using Kratos, Shimadzu with anode mono Al, pass energy 40, Current 10 mA and Voltage 12 kV. The powder X-ray diffraction (XRD) was carried out on Rigaku Smart Lab X-Ray Diffractometer (Voltage 40 kV) equipped with a rotating anode using Bragg-Brentano (BB) focusing method. Cell viability assessment was done with a microplate spectroflurometer (Multidetection microplate scanner, Dainippon Sumitomo Pharma). High-speed confocal laser-scanning microscope (CLSM, Olympus IX 81 under DU897 mode) was utilized to study NC uptake, drug transport and to differentiate live/dead population post treatment. A highly monochromatic, collimated beam of NIR range (800 nm) [Chameleon Ultra diode-Pumped Mode Locked-Sub Femtosecond Laser (Coherent 80 MHz repetition rate)] with power 2027 mW/cm^2^ (Laser power meter: VEGA, OPHIR, Japan) was utilized for all PTT and PDT experiments. The temperature variations during photothermal conversion efficiency (PCE) experiments were measured with an infrared (IR) thermometer [Thermal imager testo 881-2 (Testo AG, Germany)]. Photoacoustic imaging was performed using VisualSonics Vevo LAZR- 2100 high-frequency photoacoustic system (VisualSonics Inc., Toronto, Canada). The microCT imaging was analyzed at 45 KeV tube voltage using microCT syatem (ScanCo, Switzerland).

### Lipid-PEG functionalization, DOX loading and release study

The as prepared NCs were mixed with DSPE-PEG-Amine and were desiccated overnight. Post desiccation, the film was hydrated with PBS to yield PEG-NCs. DOX loading was performed by adding 1 mg of DOX (solubilized in DMSO) to PEG-NC suspension (10 mg/mL) and the mixture was allowed to mix in a rotator for 12 h. Post incubation, the mixture was centrifuged to collect the DOX loaded PEG-NCs from free DOX. The loading was calculated based on the absorbance of DOX bound to NCs to the absorbance of total DOX used for loading. The release of DOX from the NCs was investigated at pH 4 and pH 7. Post definite time incubation of DOX-PEG-NCs in respective pH solutions, the supernatant was collected and the absorbance of the same was measured to quantify release of DOX.

### Cell studies

MDA MB 453 (a hypermethylated cancer cell line) and MCF-7 breast cancer cell lines were purchased from Riken Bioresources. Cells were grown under ambient conditions using L-15 (MDA MB 453)/DMEM (MCF-7) medium supplemented with 10% FCS. For biocompatibility/chemotherapeutic effect analysis, 5000–10,000 cells were plated onto each well of micro well plates. Post 24 h, cells were treated with NCs (PEG-NCs/DOX-PEG-NCs) of varying concentration (0.01–1 mg) for 72 h and the cell viability was determined using commercial reagent, Alamar blue. For photothermal/photodynamic cell viability studies, cells were exposed for 2 h to 0.2/0.05 mg/mL NCs, NIR irradiated for 10/2 min respectively and their relative cell viabilities were read 24 h post treatment. For NC entry imaging assay, cells were incubated with DOX-PEG-NCs (0.1 mg/mL) for 30 min. Post-incubation, cells were imaged using confocal microscope with appropriate excitation and emission filter for DOX at different time points. For photodynamic therapy, cancer cells grown on glass base dish were exposed to PEG-NCs/DOX-PEG-NCs (0.05 mg/mL) for 30 min, post which NIR irradiation was done using 800 nm laser for 2 min. Post irradiation, cells were imaged for ROS tracer to understand the release of the same. For *in vitro* PTT, cancer cells were incubated with or without PEG-NCs/DOX-PEG-NCs (0.2 mg/mL) for 2 h and then irradiated for 10 min. Post PTT experiments, cells were stained with Calcein/PPi to differentiate live and dead population, then rinsed and viewed using confocal microscope. To study the synergistic role of chemo-PDT-PTT, we performed an experiment where cells were first exposed to NC-DOX at a concentration of 0.05 mg/mL for 4 h, and then subjected to PDT (irradiated for 2 min). Post irradiation, the cells were incubated for 4 h at ambient growth conditions after which, the cells were again irradiated for PTT (10 min) and then incubated for 16 h at ambient growth conditions. After the incubation period, the viability of cells was measured using alamar blue, the proliferation kinetics was evaluated using Ki-67 immunofluorescent staining and apoptosis was quantified using Annexin-V staining.

### Statistical Analysis

All quantitative experiments were conducted with at least three independent experiments. Statistical evaluation was performed using Origin Pro Software. Data were expressed as mean ± SE.

## Additional Information

**How to cite this article**: Poulose, A. C. *et al.* Multifunctional Cu_2−x_Te Nanocubes Mediated Combination Therapy for Multi-Drug Resistant MDA MB 453. *Sci. Rep.* **6**, 35961; doi: 10.1038/srep35961 (2016).

## Supplementary Material

Supplementary Information
