# Supplementary material for: Multifunctional Cu2−xTe Nanocubes Mediated Combination Therapy for Multi-Drug Resistant MDA MB 453
Source: Sci Rep. 2016 Oct 24;6:35961. doi: 10.1038/srep35961 (PMC5075932; doi:10.1038/srep35961)
Supplement: Supplementary Information [file srep35961-s1.doc]

**Multifunctional Cu_2-x_Te Nanocubes Mediated Combination Therapy for Multi-Drug Resistant MDA MB 453**

**Aby Cheruvathoor Poulose^1#^, Srivani Veeranarayanan^1#^, M. Sheikh Mohamed^1^, Rebeca Romero Aburto^2^, Trevor Mitcham^2^, Richard R. Bouchard^2^, Pulickel M. Ajayan^3^, Yasushi Sakamoto^4^, Toru Maekawa^1^, D. Sakthi Kumar^1^***

^1^*Bio Nano Electronics Research Centre, Graduate School of Interdisciplinary New Science, Toyo University, Kawagoe, Japan- 350-8585*

^2^*Department of Imaging Physics, University of Texas MD Anderson Cancer Center, Houston, TX 77054, USA*

^3^*Department of Material Science and NanoEngineering, Rice University, 6100 Main Street, Houston, TX 77005, USA*

^4^ *Biomedical Research Centre, Division of Analytical Science, Saitama Medical University, Saitama 350-0495, Japan*

**SUPPORTING INFORMATION**

**Corresponding author***

Prof. D. Sakthi Kumar,

Ph: 81-492-39-1636

Fax: 81-492-34-2502

E-mail: [sakthi@toyo.jp](mailto:sakthi@toyo.jp)

# Both authors contributed equally.

**
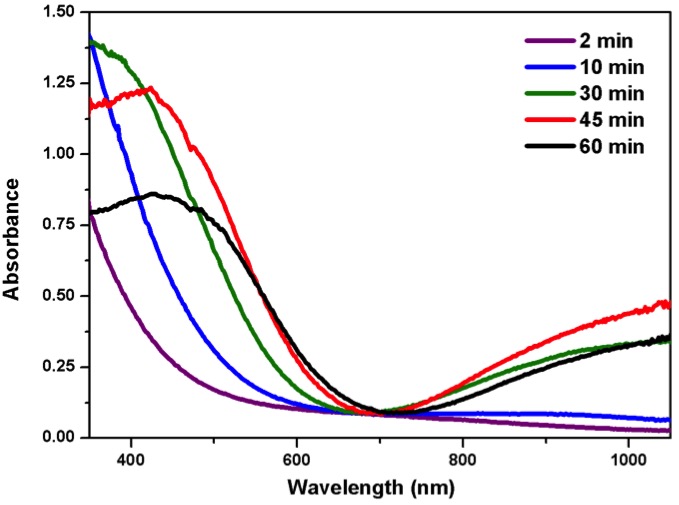
**

**Figure S1:** UV-Vis NIR absorption of NCs synthesized at various reaction times.

**
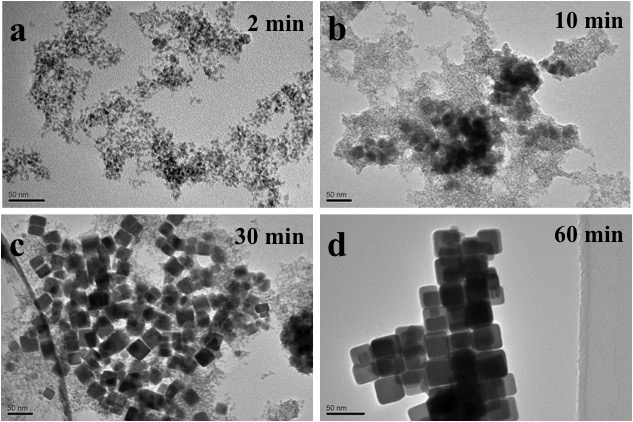
**

**Figure S2:** TEM images of representative NCs synthesized at various reaction times.

**
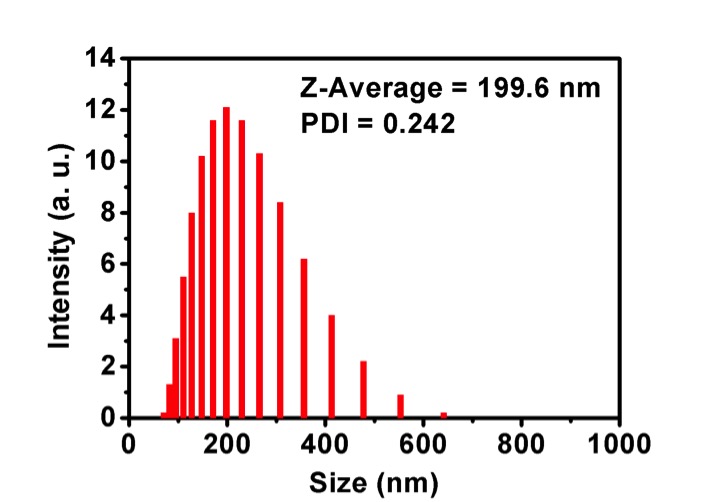
**

**Figure S3:** Hydrodynamic diameter of PEG-NCs.

**
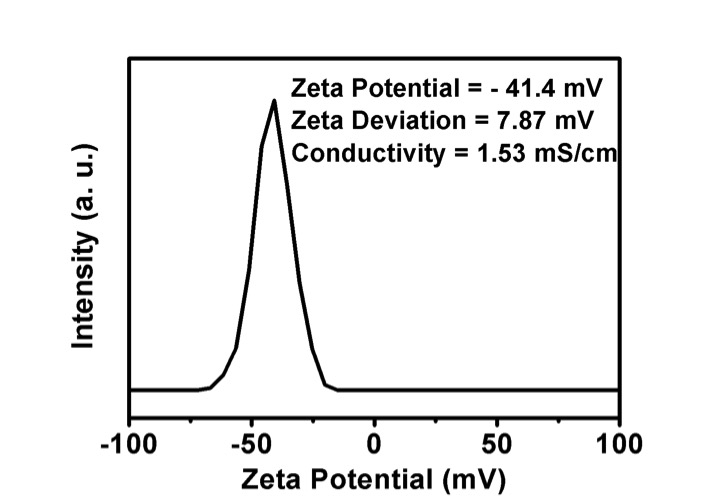
**

**Figure S4:** Zeta potential of PEG-NCs.

**
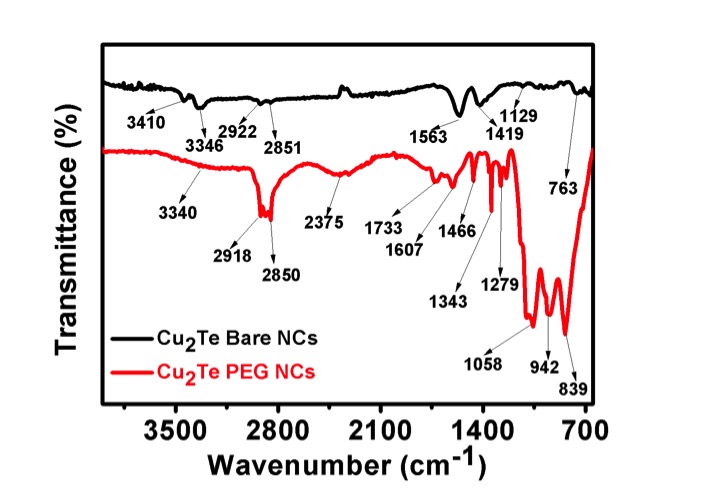
**

**Figure S5**: FT-IR analysis of PEG-NCs.


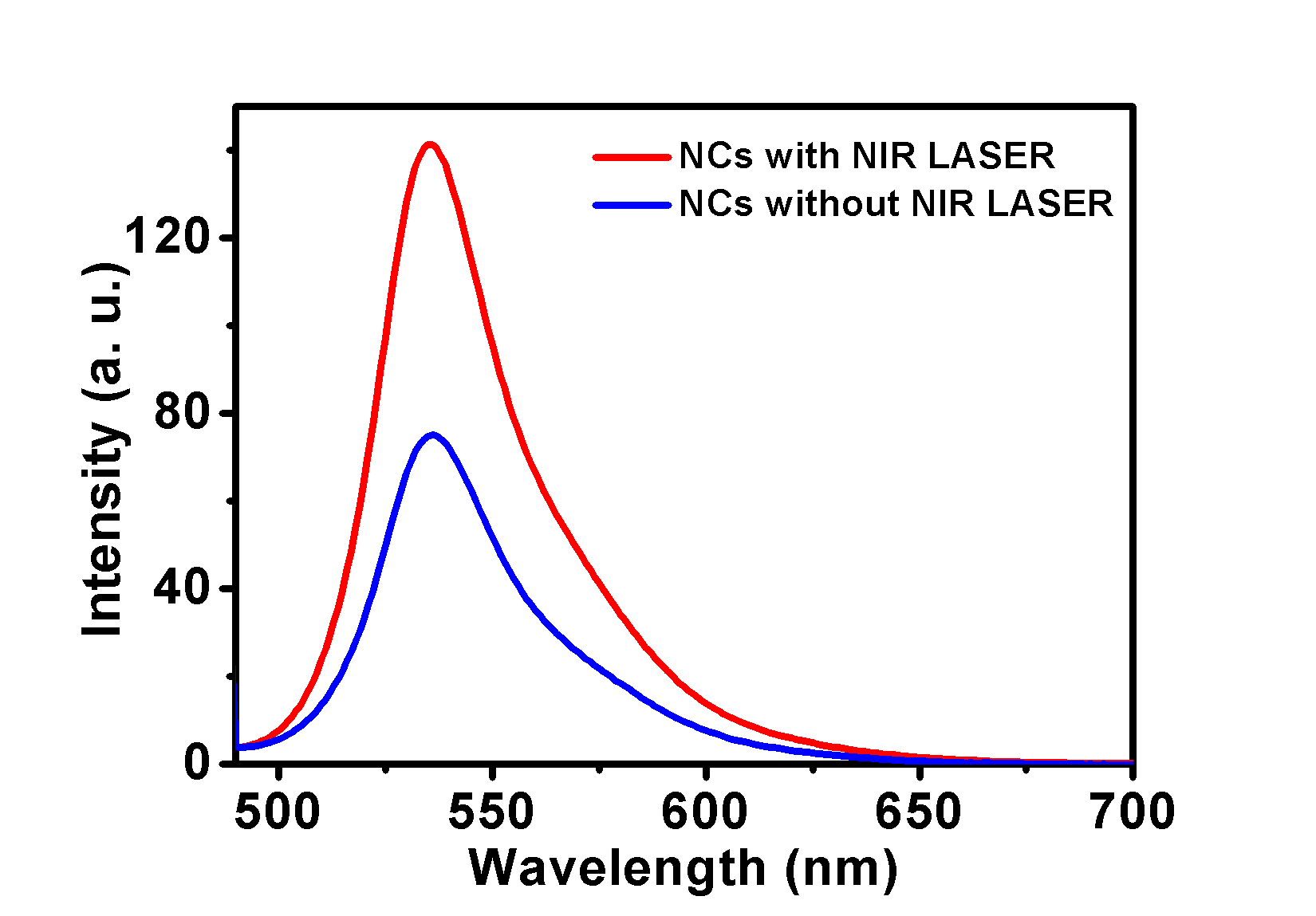


**Figure S6:** PL spectra of DCF – a ROS tracer in the aqueous suspension of NCs that are either exposed/not to NIR irradiation.

**
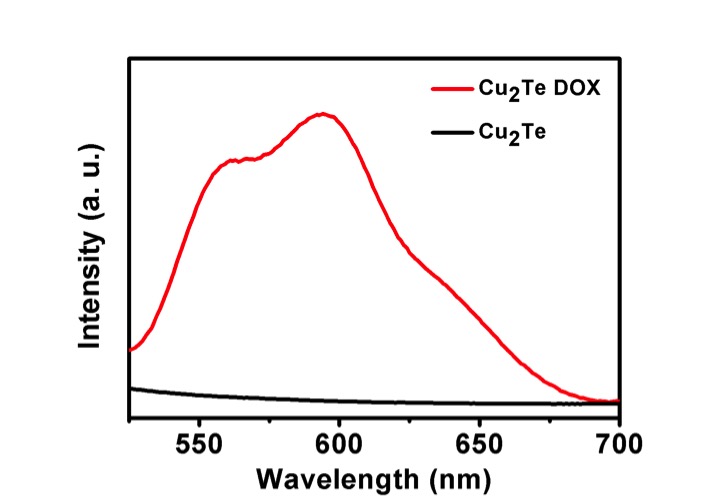
**

**Figure S7:** PL emission spectra of PEG-NC-DOX conjugate in comparison to PEG-NCs.

**
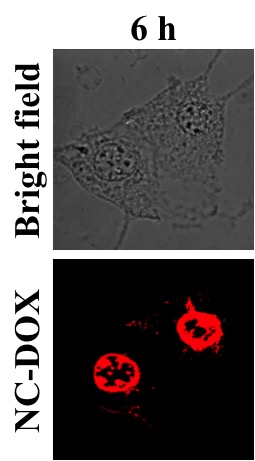
**

**Figure S8:** Nuclear localization of DOX post exposure of PEG-NC-DOX exposure in MCF-7 cancer cell.
